# Mycoplankton Biome Structure and Assemblage Processes Differ Along a Transect From the Elbe River Down to the River Plume and the Adjacent Marine Waters

**DOI:** 10.3389/fmicb.2021.640469

**Published:** 2021-04-23

**Authors:** Yanyan Yang, Stefanos Banos, Gunnar Gerdts, Antje Wichels, Marlis Reich

**Affiliations:** ^1^Molecular Ecology Group, University of Bremen, FB2, Bremen, Germany; ^2^Alfred Wegener Institute Helmholtz Centre for Polar and Marine Research, Biologische Anstalt Helgoland, Helgoland, Germany

**Keywords:** aquatic fungi, estuary, QPE analysis, salinity gradient, Chytridiomycota, shallow freshwater, dispersal, habitat

## Abstract

Rivers are transport systems and supply adjacent ecosystems with nutrients. They also serve human well-being, for example as a source of food. Microorganism biodiversity is an important parameter for the ecological balance of river ecosystems. Despite the knowledge that fungi are key players in freshwater nutrient cycling and food webs, data on planktonic fungi of streams with higher stream order are scarce. This study aims to fill this knowledge gap by a fungi-specific 18S ribosomal RNA (rRNA) gene tag sequencing approach, investigating mycoplankton diversity in the Elbe River along a transect from shallow freshwater, to the estuary and river plume down to the adjacent marine waters (sections of seventh stream order number). Using multivariate analyses and the quantitative process estimates (QPEs) method, questions (i) of how mycoplankton communities as part of the river continuum change along the transect, (ii) what factors, spatial and environmental, play a role, and (iii) what assembly processes, such as selection or dispersion, operate along the transect, were addressed. The partitioning of mycoplankton communities into three significant distant biomes was mainly driven by local environmental conditions that were partly under spatial control. The assembly processes underlying the biomes also differed significantly. Thus, variable selection dominated the upstream sections, while undominated processes like ecological drift dominated the sections close to the river mouth and beyond. Dispersal played a minor role. The results suggest that the ecological versatility of the mycoplankton communities changes along the transect as response, for example, to a drastic change from an autotrophic to a heterotrophic system caused by an abrupt increase in the river depth. Furthermore, a significant salinity-dependent occurrence of diverse basal fungal groups was observed, with no clade found exclusively in marine waters. These results provide an important framework to help understand patterns of riverine mycoplankton communities and serve as basis for a further in-depth work so that fungi, as an important ecological organism group, can be integrated into models of, e.g., usage-balance considerations of rivers.

## Introduction

Rivers are unique ecosystems that strongly influence adjacent terrestrial ecosystems and coastal waters through their nutrient input. Furthermore, they serve human well-being by acting as food source, drinking water reservoirs, or waterways, among other things. It is therefore all the more important that human use does not disturb the ecological balance of the river (Friberg et al., 2011). Different scenarios are often modeled for usage-balance considerations (Regier and Kay, 1996; Schöl et al., 2014; Schiemer et al., 2020). Beside physical properties or topology, microbial diversity is a non-negligible parameter, as microorganisms are key players of riverine carbon and nutrient cycles and stand at the beginning of food web chains. Numerous inventories of riverine bacterioplankton communities exist targeting rivers and river sections of different stream order numbers (Selje and Simon, 2003; Read et al., 2015; Huber et al., 2020). In contrast, the existing studies on fungi mainly focused on streams of lower order numbers and/or focused on individual fungal groups (Wood-Eggenschwiler and Bärlocher, 1983; Lecerf and Chauvet, 2008), eukaryotic communities (Simon et al., 2015; Lu et al., 2020; Shi et al., 2020), or habitats other than the water body (Liu et al., 2015; Chen et al., 2020). This is a major contradiction to the knowledge that mycoplankton are key components of ecosystem functioning in all freshwater systems. For example, pelagic fungi possess an extremely diverse enzymatic repertoire (Zemek et al., 1985; Chrismas and Cunliffe, 2020), are involved in various nutrient cycles, and exert top–down control on phyto- and zooplankton (Barron, 2004; Rasconi et al., 2011; Frenken et al., 2017). Grossart et al. (2019) outlined the following two cycles as important aquatic ecosystem services of pelagic fungi: *Mycoflux*, which describes the fungal decomposition and provision of organic material for other organisms; *Mycoloop*, in which zoosporic fungi represent an important trophic link between phytoplankton and zooplankton; by infecting large, grazing-resistant phytoplankton species, the fungus can use the host’s nutrients and organic material to form new zoospores, which can then be grazed by zooplankton.

Lotic systems differ from other freshwaters in that they form a continuum of time and space during the transition from the upper course of the river to the river mouth, which finally fades out in the river plume. During the passage of the river, the water masses are exposed to different influences. For example, the adjacent landscape structure, anthropogenic activities or physical streams have an effect on the site-specific water chemistry, residence time, and flow velocity (Friberg et al., 2011; Geerts et al., 2017). Thus, the biota occurring in the water body are exposed to physiochemical and hydrological gradients. This phenomenon of a downstream gradient that biota have to cope with is referred to as the “river continuum concept” (Vannote et al., 1980). One of the strongest gradients around the river mouth is salinity, which influences the presence and abundance of many organisms (Attrill, 2002; Telesh et al., 2011), including fungi (Livermore and Mattes, 2013). In addition to environmental gradients, the water transport along a given longitudinal direction also influences the composition of the biotic communities. The dispersal strategy is therefore an important variable for the occurrence of individual taxa. Aquatic fungi possess very different dispersal strategies: Early diverging lineages display morphological adaptations in the form of zoospores or amoeboid spores, while (James and Berbee, 2012; Powell and Letcher, 2014; Letcher et al., 2015) some ascomycetes form spore appendages, with which they can attach to particles (Jones, 2006). Other possibilities include resistant spores, fragments of hyphae, the whole thallus, and as passenger on/in particles or host tissue (Kohlmeyer and Kohlmeyer, 1979; Gleason and Lilje, 2009).

Given that fluvial fungal communities are important players in ecological processes (Krauss et al., 2011), can hold human–pathogenic members (Ulfig et al., 1997), but also are source for the discovery of new antibiotics or biotechnologically relevant natural products (Svahn et al., 2012; Grossart and Rojas-Jimenez, 2016), it is of critical importance to gain a better understanding of the spatial patterns of fluvial fungal diversity, their main driving forces, and their persistency in the face of changing environmental conditions. Therefore, we addressed here the following questions: (i) To what extent do mycoplankton communities change, as part of the river continuum, from shallow freshwater through the estuary down to the Elbe River plume and adjacent marine waters? (ii) What are the driving forces? (iii) Which assemblage processes affect the mycoplankton communities, and do they differ along the sampled transect? (iv) Since the transect spans a sharp salinity gradient, how does this affect the fungal distribution and abundance with eye mark on basal fungal lineages. We hypothesized that communities are segregated along fresh, brackish, and marine water types and due to high heterogeneity in environmental parameters across the transect that communities are composed primarily by variable selection.

## Materials and Methods

### Sampling and Measurements of Environmental Parameters

**FIGURE 1:**
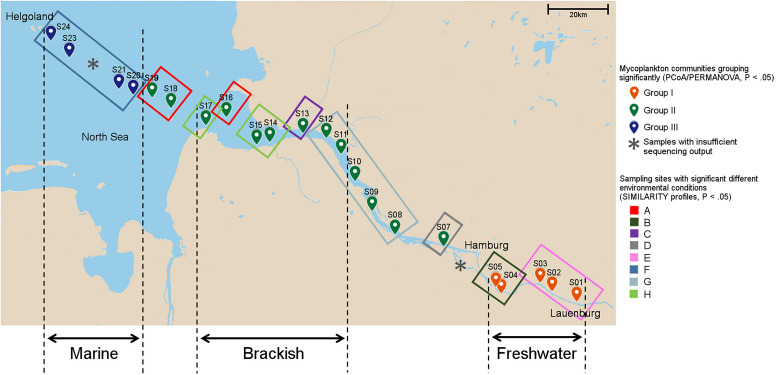
Sampling sites and local environmental conditions. The environmental heterogeneity between sites was analyzed with similarity profiles. Sample positions encircled with the same color in the map indicate similar environmental conditions (*P* < 0.05). Mycoplankton communities from individual sampling sites that show similar phylogenetic composition as identified by principal component analysis (PCoA)/permutational multivariate ANOVA (PERMANOVA) (*P* < 0.05, see Figure 2B), have the same color for the sampling point. Notably, the grouping differed from the water type zonation of fresh-, brackish, and marine waters. In the Elbe River, the end member of the freshwater can shift depending on the strength of the water runoff. Thus, to test a water-type-specific distribution of the basal fungi (see Figure 5), only samples that fall into one water type throughout the year were used. “X” indicates samples that were excluded from further analyses due to low sequence output.

The study was conducted over a progressively increasing salinity gradient spanning from the shallow freshwater area of the Elbe River into the Elbe estuary, the river plume, and the transition zone with marine water in the North Sea. The curved transect line, following the course of the river and lying within the seventh Horton–Strahler order stream section of the Elbe (Schmidt et al., 2020), traversed in total 24 sampling stations starting at the city of Lauenburg (Germany, 53°22′11.60″N, 10°33′8.37″E) and ending close to the island of Helgoland in the German Bight (Germany, 54°09′06.1″N, 7°53′30.1″E) (Figure 1 and Supplementary Table 1). The total length of the curved line transect was 217.8 km, measured as the cumulative water channel distance. Sampling was carried out from the August 4–6, 2015 during the tidal water runoff to keep sampling conditions stable and avoid influence from incoming North Sea water. Surface water was sampled beside the fairway at a water depth of ∼1 m into a sterile 10-L bottle (Nalgene, Germany) from onboard of the research vessel Uthörn or offboard from the coast line. Two liters of the water was filtered on a 0.22-μm polyethersulfone (PES) membrane (Merck, Darmstadt, Germany) using a peristaltic pump and stored until further treatment at −20°C.

At each of the 24 sampling points, additional water was collected to measure 10 environmental parameters, namely, salinity, pH, water temperature, nitrate, nitrite, ammonium, phosphate, silicate, chlorophyll *a*, and dissolved organic matter (DOC) (Supplementary Table 1; PANGAEA dataset: Reich et al., 2021). A detailed description of the sampling procedure and analyses can be found by Lucas et al. (2016). Incomplete data fields for salinity (samples 1-8) were filled up by searching the appropriate values on the data portal “Fachinformationssystem (FIS)” of the FGG (FlussGebietsGemeinschaft, Magdeburg, Germany) Elbe (Flussgebietsgemeinschaft_(FGG)_der_Elbe, 2020) if possible. Furthermore, based on literature values, which confirmed the measured ones, samples were grouped into the three water types of fresh, brackish, and marine waters following the definition of Remane and Schlieper (1972) with < 0.5, > 0.5 - < 30, and > 30 practical salinity units (PSU), respectively, as water-type specific salinity range. Not all samples could be considered in this classification because the freshwater boundary of the Elbe shifts depending on the season and the associated intensity of water runoffs (Dähnke et al., 2008). In summer, it can be located at the level of the port of Hamburg (145 km to the river mouth) or, in the case of strong water runoffs, at the level of Glücksstadt (85 km to the river mouth). For this reason, we used samples whose position lay within one water type over the whole year (Bergemann, 1995; Amann et al., 2012). Thus, samples 1-5, 11-17, and 20-24 were considered as fresh, brackish, and marine water samples, respectively. Furthermore, waterway deepening in rivers has significant impact on river ecosystems (Manap and Voulvoulis, 2016). To measure a possible indirect impact of dredging activities on the fungal community composition, we took the depth of the sampling stations as proxy. Incomplete data fields (samples 1-16) were filled by using the digital relief model of the river, which is the DGM-W 2010 Unter-und Außenelbe data (Digitales Geländemodell des Wasserlaufes) provided by the Zentrales Datenmanagement (ZDM), Küstendaten, of the Wasserstraßen- und Schifffahrtsverwaltung des Bundes (DGM-W_Unter_und_Außenelbe, 2010) (Supplementary Table 1).

### DNA Extraction, Sequencing, and Bioinformatics

DNA was extracted using the Power Water DNA Isolation kit (MO BIO Laboratories, Carlsbad, CA, United States) according to the manufacturer’s instruction. PCR reactions were performed using the fungi specific 18S ribosomal RNA (rRNA) primer pair nu-SSU-1334-5′/nu-SSU-1648-3′ (Vainio and Hantula, 2000), recently shown to be the best performing primer pair on aquatic fungal assemblages (Banos et al., 2018, 2019). Additionally, four annealing blocking oligonucleotides with a 3′-amino linker C6 modification were added to the PCR reaction to prevent coamplification of the abundant eukaryotic groups of Alveolata, Rhizaria, Stramenopiles, and *Telonema* (Banos et al., 2018). PCR, library preparation, and sequencing was performed at LGC Genomics GmbH (Berlin, Germany). All sequencing reactions were done with the IlluminaMiseq Reagent Kit v3 for 2 × 300 bp reads (Illumina, Berlin, Germany) following the manufacturer’s instructions.

Sequences were analyzed with a phylogeny-based approach following the pipeline of Banos et al. (2020). Shortly, quality-controlled sequence reads were passed on for classification. In a first step, reads were incorporated into the backbone alignment of the non-redundant SILVA database SSURef_132 (Quast et al., 2013) using the SINA aligner v1.2.11 (Pruesse et al., 2012) with the default settings. This step includes the classification of the query sequences by using the 10 most similar sequences as provided by the alignment and applying the least common ancestor rule (LCA) with a 95% sequence similarity threshold. Only sequences that were classified in this way as fungi were further clustered into operational taxonomic units (OTUs) based on a 98% sequence similarity using the CH-HIT-EST tool within the CD-HIT software v4.6 (Li and Godzik, 2006; Fu et al., 2012). The final classification of the OTUs’ reference sequences was carried out by inserting them over phylogenetic placement into the fungal phylogenetic reference tree (Yarza et al., 2017). Prior to the insertion, the fungal reference tree was enriched by 254 fungal 18S rRNA gene sequences of the SILVA dataset SSURef_128 not yet present in the tree, by 210 reference sequences of so-far unrecognized soil-inhabiting order-level clades described by Tedersoo et al. (2017) and by 79 sequences of newly identified basal fungal taxa (Seto et al., 2020; Simmons et al., 2020; and uploaded sequence data on INSDC accession numbers: KJ668047–KJ668085). After inserting the generated sequences, the tree was inspected for novel diversity clades formed by at least five OTUs represented by environmental sequences. In this case, the lowest possible taxonomic level was transferred and the word “clade,” and a number was added. For example, if a novel diversity clade was formed on the branch of the Chytridiomycota, it got the name “Chytridiomycota clade x,” while a clade on the branch of the Chytridiomycetes got the name “Chytridiomycetes clade y.” In case of several new clades formed on the same taxon level, the clades were numbered in ascending order (Supplementary Figure 1).

For further analyses, a subcommunity of all abundant OTUs was formed containing those whose relative sequence abundance was summed up to 90% of the one of the total community. Beside the phylogenetic placement into the reference tree, sequences of the abundant OTUs were compared to sequences in the non-redundant nucleotide collection in GenBank of the National Center for Biotechnology Information (NCBI) database using BLASTN 2.11.0 + with the default settings but excluding environmental sequences. The best BLAST hits were selected based on E < 1e^–130^, query coverage >99%, and sequence identity ≥99%. In case of several best BLAST hits, all were documented. Finally, the primary scientific literature was searched to assign, if possible, a nutrition mode to the identified taxa. The BLASTn step was mainly performed to gain information on the potential nutritional mode of the taxa rather than for general taxonomic classification on a low taxonomic level, as BLASTn results on species level performed on short 18S rRNA gene sequences have to be handled with care (Reich and Labes, 2017).

### Statistics

If not differently stated, the statistical analyses were carried out within the *R environment* v4.0.2 (R Core Team, 2015). Rarefaction curves were generated with the “iNEXT” function of the R package *iNEXT* (Hsieh et al., 2016). Next, prior to any calculation, OTU counts were subjected to Hellinger transformation (Bhattacharyya, 1943) and contextual data to z-scoring transformation (Clark-Carter, 2014). Environmental factors were checked for collinearity using a Spearman rank correlation test and adjusting the *P* values with the false discovery rate (FDR) method (Benjamini and Hochberg, 1995), and highly correlating factors were removed (Supplementary Table 2). To identify which mycoplankton communities from the different sampling sites own a similar phylogenetic composition, a distance-based principal component analysis (PCoA) was run using generalized Unifrac (GUnifrac) distance values as input. The significance of the observed sample clustering in the PCoA was tested by permutational multivariate ANOVA (PERMANOVA) (FDR adjusted *P* < 0.05). The correlation of the environmental variables with the first two axes of the PCoA was calculated by the Pearson correlation coefficient with default settings between sample scores on each axis and each of the environmental variables (FDR, adjusted *P* < 0.005, R^2^ > 0.5, score > | 0.7|). All these steps were calculated with the R packages *phylosec* (McMurdie and Holmes, 2013) and *GUniFrac* v1.1 (Chen et al., 2012) and *pairwiseAdonis* (Martinez Arbizu, 2020).

To identify samples with a similar environmental profile, a non-hierarchical clustering based on k-means and coupled to similarity profile test (SIMPROF) was performed using PRIMER v7 (Primer-e, 2017) on the basis of the z-transformed environmental factor matrix. The significance level for SIMPROF was set to 5% and performed with 999 permutations to define the optimal number of k-groups (between 2 and 10) to describe the clustering of the samples, which is based on maximizing R.

The sample groups well separated in the PCoA and identified as significantly different by PERMANOVA were further inspected. Thus, for each group, average OTU richness (Chao1) and diversity (Shannon) was calculated using the “estimate_richness” function in the package *phyloseq* (McMurdie and Holmes, 2013). Additionally, phylogenetic diversity (PD) was calculated using the program PHYLOCOM v4.2 (Webb et al., 2008). Significance in α-diversity between the different sample groups was tested with the Tukey’s *post hoc* test [Tukey honestly significant difference (HSD)] with the default settings in the package *states* (Gleditsch and Ward, 1999). Correlations of the abundant OTUs with environmental factors were tested by Pearson rank order correlations. Furthermore, PERMANOVA was applied to test if the salinity value of 8 PSU significantly separates fungal communities.

To check if an impact of the geographical distance on the phylogenetic dissimilarity exists, a distance-decay analysis was carried out applying linear regression (*n* = 231; *P* < 0.05) using the “lm” function of R. As this showed to be significant, the power of control of spatial factors on the fungal community variation was further tested. Thus, a spatial eigenfunction was carried out using the “distance-based Moran’s eigenvector maps” (dbMEM) function of the R package *adespatial* (Dray et al., 2017) to calculate eigenfactor and eigenvalues. As our sampling was not a standard sampling situation (e.g., not a straight transect line), the truncated distance matrix was generated following the example of Brind’Amour et al. (2005) but not looping the sample sites at the end. Eigenfunctions with a positive eigenvalue were tested for significance (*P* ≤ 0.05) by distance-based redundancy analysis (dbRDA)-based forward selection [function “ordistep” in the R package *vegan* (Oksanen et al., 2013)]. Based on a scalogram using the Moran’s I coefficient as ordinate, significant distance-based Moran’s eigenvector map (dbMEM) eigenfunctions were defined to different scaling submodels. Next, to explain the partitioning of the observed variation in the mycoplankton community between the two components, spatial and environmental, a dbRDA-based model was also built on the environmental factors using forward selection. Finally, the different spatial submodels and the first three best models on the environmental parameters were independently used as input for variation partitioning analysis (VPA) by dbRDA using the “varpart” function in the R package *vegan* (Oksanen et al., 2013).

To estimate which ecological processes influence the fungal community at the different sampling sites, the statistical framework of Stegen et al. (2015) incorporated in the quantitative process estimates (QPEs) method was applied. This method considers five different assembly processes, namely, variation selection, homogenizing selection, dispersal limitation, homogenizing dispersal, and undominated processes, which are a lack of dominance between selection and dispersal. This approach requires significant phylogenetic signals, which are used for interpretation. First, the phylogenetic turnover of communities between the diverse sites was calculated as β-mean nearest taxon distance (MNTD) metric. Next, a null expectation was tested, meaning that ecological selection was not the primary factor of compositional differences by randomly reshuffling the OTUs over the tips of the phylogenetic tree. Significance was evaluated via the nearest taxon index (β-NTI) expressing the differences between the observed β-MNTD and the mean of the null distribution in units of SD. In the case of βNTI > | 2|, a significant deviation from the null expectation exists, and variable (βNTI > 2) or homogenizing (βNTI < –2) selection is responsible for the differences observed. If the observed βNTI is not due to selection (| βNTI| < 2), it can be due to low or high rates of dispersal or undominated process (Stegen et al., 2012). To distinguish among these possibilities, the Raup–Crick metric (βRC_bray_) (Chase et al., 2011) was calculated with βRC_bray_ > 0.95 and βRC_bray_ < –0.95, indicating dispersal limitation and homogenizing dispersal, respectively, while βRC_bray_ < | 0.95| reflects an undominated process. All calculations were done with the R code provided by Stegen et al. (2013) in GitHub^1^. Significant differences in the assembly processes among the sampling groups were calculated by Tukey HSD.

Another important question arose from the strong salinity gradient that spans the river section under investigation. The water’s salinity seems to be an important factor in the distribution and occurrence of aquatic basal fungi (Livermore and Mattes, 2013) (basal fungi include Chytridiomycota, Rozellomycota, and novel diversity clades on the branch of basal fungi). Samples 1–5, 11–17, and 20–24 were considered as above described as freshwater, brackish, and marine samples, respectively. (Note that in some cases, this grouping is not equal to the sample grouping found by PCoA/PERMANOVA and has solely been used to identify salinity-driven water-type-specific occurrence of the basal fungal lineages). Significant distribution (using the frequency) of the diverse basal fungal lineages over the three water types was tested with the Tukey HSD.

## Results

### Taxonomic Community Composition

Fungal sequence reads (647,568) were generated and clustered into 913 fungal OTUs. Prior to further analyses, the two samples, 6 and 22, were removed due to low sequence output. All rarefaction curves levelled off reaching a plateau, indicating sufficient sequencing depth to capture most of the mycoplankton diversity (Supplementary Figure 2). OTUs were phylogenetically classified into six fungal phyla, 15 subphyla, 32 classes, and 55 orders. The phylogenetic approach led to the recognition of novel diversity forming 16 new clades, seven within the Chytridiomycota, seven within the Rozellomycota, and two branched along the basal fungi (Supplementary Figure 1). Three of the Chytridiomycota clades and five of the Rozellomycota clades clustered together with reference sequences of novel diversity groups recognized by Tedersoo et al. (2017). Additionally, abundant OTUs within the Chytridiomycetes clade 01 and Basal Fungi clade 02 had supporting BLASTn hits with sequences of *Chytridium polysiphinae* and an uncultured Rozellomycota, respectively (Supplementary Table 3).

**FIGURE 2:**
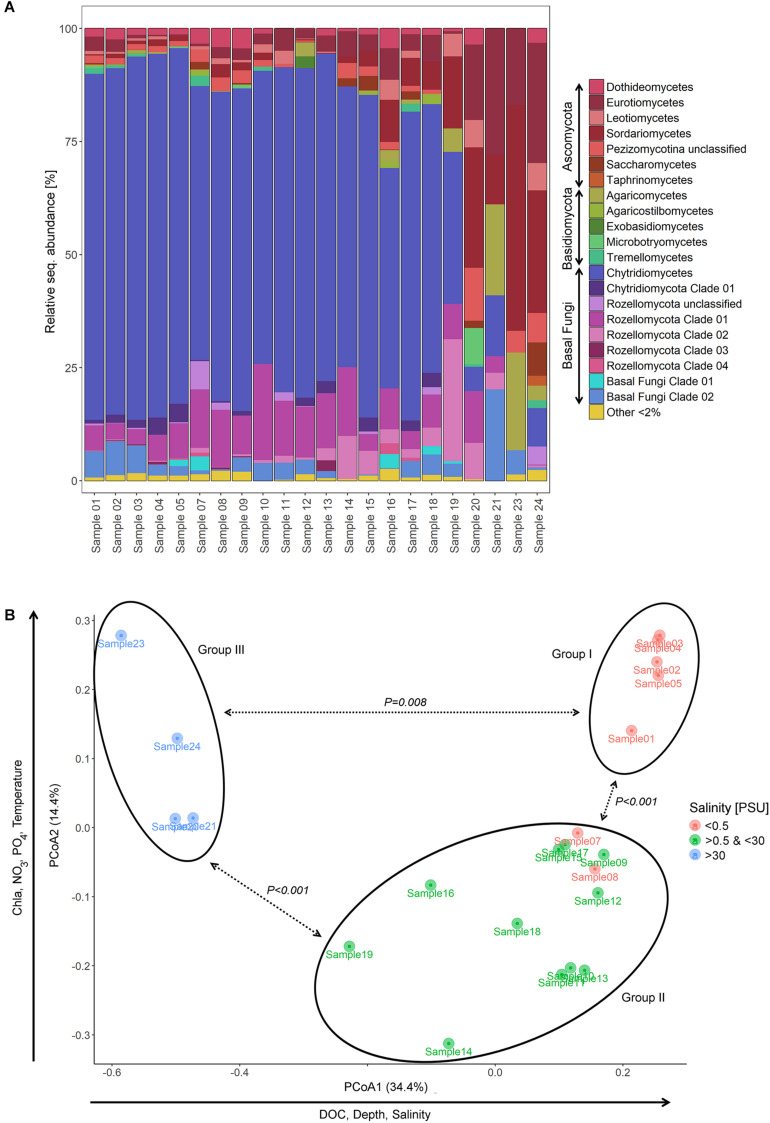
Partitioning of the mycoplankton communities. **(A)** Bar chart showing the relative sequence abundance of mycoplankton classes and novel diversity clades of samples taken on a transect of the lower reaches of the Elbe River including samples from the shallow, freshwater (samples 1–3) through the estuary (samples 4–17), to the river plume (samples 18–19), and the adjacent marine waters of the North Sea (samples 20–24). For information on taxonomic classification, see Supplementary Figure 1. **(B)** Principal component analysis (PCoA) ordinating mycoplankton communities based on their phylogenetic composition (Generalized UniFrac). Communities clustered in three significant different groups (PERMANOVA, *P* values see graph). Group I included samples of the shallow, freshwater zone of the Elbe transect; group II, samples taken at locations with pronounced river bed depth due to dredging activities; and group III, samples of the outer river plume. PSU, practical salinity unit.

From the shallow freshwater area of the Elbe River to the marine environment, the relative abundance of Chytridiomycota decreased from 84.6% (sample 1) to 0% (sample 24), while Dikarya OTUs showed an opposite pattern, increasing from 7.5% (sample 1) to 94.7% (sample 24). Rozellomycota taxa were represented in all samples except one with up to 34.8% (sample 19) of the relative sequence abundance and were mainly accounted for by Rozellomycota clade 01 (Figure 2A).

Nineteen OTUs were defined as abundant, from which 13 were classified as Chytridiomycota, 5 as Ascomycota, and 1 as Basal Fungi clade 02. In the BLASTn analysis, all of them showed high sequence similarity to sequences annotated to species level. For all of the matched sequences except one, literature screening allowed the identification of the taxon’s nutritional mode (Supplementary Table 3). The most abundant OTU was OTU SMBZZZZ9 from the order Zygophlyctidales, representing up to 86.2% of the relative sequence abundance in the samples and being present in all samples with two exceptions (samples 20 and 23) (Supplementary Tables 3, 4). All Ascomycota OTUs showed highest frequency in the sample 24 located already beyond the river plume in marine waters (sample 24: position most close to the island of Helgoland). In contrast, nine of the other OTUs were highly frequent in the shallow freshwater area upstream of Hamburg (samples 1–5), but all were also present in the downstream area of Hamburg (samples 7–24). Here, two different pattern types were observed. The first one was a continuous OTU presence over numerous adjacent sampling sites (>4) with sometimes further occurrence at other sampling sites but then usually with a lower frequency (84.2% of all abundant OTUs). The second pattern was defined by OTUs with presence at sampling sites with often high frequency but not in more than four adjacent sampling stations (15.8%) (Supplementary Figure 3).

### Fungal Community Partitioning and Impact of Environmental Factors

PCoA ordinated the samples based on their phylogenetic diversity into three different sample groups with axis 1 explaining 34.4% and axis 2 14.4% of the observed separation (Figure 2B). Samples 1–5 grouped together (group I) and included samples of the shallow, freshwater water region upstream of Hamburg, with two samples taken at sites with tidal influence. Samples 7–19 formed a distant group (group II), which comprised samples of sites with fairway adjustment through dredging. Hereby, both freshwater and brackish water samples were represented. Sample group III was formed by samples 20–24, which were defined as marine based on the salinity values. The three sample groups differed significantly from each other [PERMANOVA (F value, R^2^, FDR-adjusted *P* value), groups I and II: 9.3, 0.37, 0.003; groups I and III: 57.5, 0.89, 0.006; groups II and III: 30.1, 0.67, 0.003] (Figures 1, 2B).

Environmental parameters were tested for collinearity using the Spearman rank order correlations identifying strong collinearity (FDR adjusted *P* < 0.05, R^2^ > 0.5) mainly between NO_2_ and NH_4_ and between salinity, depth, and temperature (Supplementary Table 2). Prior to any analysis, we excluded NO_2_ and NH_4_, as the values of NO_3_, NO_2_, and NH_4_ depend in their dynamics on each other in the Elbe River (Amann et al., 2012; Sanders et al., 2018), while the impact of salinity, depth, and temperature was analyzed separately (for example in different models). Pearson’s correlation coefficient identified salinity (*P* = 0.001, R^2^ = 0.79), DOC (*P* = 0.001, R^2^ = 0.62), and depth (*P* = 0.001, R^2^ = 0.52) as explaining environmental variables for the sample separation along the axis 1, and PO_4_ (*P* = 0.001, R^2^ = 0.66), NO_3_ (*P* = 0.001, R^2^ = 0.66), temperature (*P* = 0.001, R^2^ = 0.76), and chlorophyll *a* (*P* = 0.001, R^2^ = 0.70) as the one for axis 2 (Figure 2B and Table 1). The sampling strategy had no impact on observed community differences (Tukey HSD, *P* > 0.05). Similarity profiles on environmental factors identified a wide environmental heterogeneity over the different sampling sites clustering the samples into eight distant groups (*P* < 0.05). Compared to the sample groups defined by PCoA and PERMANOVA, sample group I falls into regions with two different overall environmental conditions, sample group II into five, and only the marine sample group III showed relative homogeneous environmental conditions over the four sample locations (Figure 1).

**TABLE 1:** Environmental factors with significant correlation with the first two principal component analysis (PCoA) axes, as tested by Pearson correlation coefficient analysis showing the FDR-adjusted *P* < 0.005 and R^2^ > 0.5 for one of the two axes.

	Axis 1	Axis 2	R^2^	*P*
PO_4_	0.25	−**0.96**	0.66	0.002
NO_3_	0.69	−**0.71**	0.65	0.002
Chl *a*	0.44	**0.89**	0.69	0.002
Salinity	−**0.98**	–0.16	0.79	0.002
Temperature	0.59	**0.80**	0.76	0.002
DOC	**0.83**	–0.55	0.62	0.002
Depth	−**0.97**	–0.22	0.51	0.003

*In bold, factors significantly related with one of the two axes.*

Pronounced differences were observed in the correlations among the abundant OTUs of Ascomycota and Chytridiomycota with environmental parameters. Most Chytridiomycota OTUs showed negative correlations with depth and salinity and positive ones with temperature, chlorophyll *a*, and silicates. DOC was also among the positive correlated factors but less pronounced. Ascomycota OTUs, however, were negatively correlated mainly with silicate and nitrate (*P* < 0.05) (Supplementary Table 5).

As the value of 8 PSU is stated in the literature as the value separating organismic communities along salinity gradients, it was tested with PERMANOVA if a PSU of 8 also applies for the mycoplankton assemblage in this study. Indeed, when tested only for this factor, fungal communities significantly separated at the 8 PSU with samples 1–12 and 13–22 in the two different groups (*F* value = 10.04, R^2^ = 0.33, FDR-adjusted *P* < 0.001).

The comparative analysis as PCoA including communities of this study and the one of Banos et al. (2020) monitoring mycoplankton communities at Helgoland Roads over a year showed a grouping of samples 20–24 (marine samples) with the samples of Banos et al. (2020), while the sample group structure of groups I and II remained intact and well separated from the rest (Supplementary Figure 4).

### α-Diversity

Sample group I stood out with the highest OTU richness and phylogenetic diversity among all identified sample groups with significant higher Chao1 and PD values of 395.9 ± 138.7 and 12.3 ± 2.7, respectively (Tukey HSD, permutations = 999, *P* < 0.001). Sample group III showed lowest Chao1 and PD values with 37.5 ± 35.4 and 1.8 ± 1.3, respectively. In contrast, no significance was found in Shannon diversity among sample groups ranging from 2.1 to 1.9 (Table 2).

**TABLE 2:** α-Diversity for the three identified samples groups (see Figure 2).

α-diversity	Group I	Group II	Group III	Significant difference (*P* < 0.001)
Chao1	395.9 ± 138.7	109.1 ± 53.8	37.5 ± 35.4	GI–GII, GI–GIII
Shannon	2.0 ± 0.58	2.1 ± 0.58	1.9 ± 0.6	
PD	12.3 ± 2.7	4.7 ± 1.8	1.8 ± 1.3	GI–GII, GI–GIII

*Taxon richness (Chao1), diversity (Shannon), and phylogenetic diversity (PD) was calculated for each group (± SD), and significance between groups was tested with the Tukey’s post hoc test (*P* < 0.001).*

### Impact of Spatial and Environmental Factors on Community Assemblage

The phylogenetic dissimilarity among fungal communities increased gradually with distance of the sampling sites. The relationship was significant as shown by linear regression (*n* = 231, *P* < 0.001, r^2^ = 0.339) (Supplementary Figure 5). dbMEM identified 21 eigenvectors, from which seven were positive along Moran I. Forward selection attested four out of all positive eigenvectors a significant impact on the fungal assemblage (Supplementary Table 5). Based on the scalogram of Moran’s I, two different submodules (small and broad local scale) were defined (dbMEM1 and 2 as broad-scale factors; dbMEM3 and 5 as small-scale factors) (Supplementary Figure 6).

**FIGURE 3:**
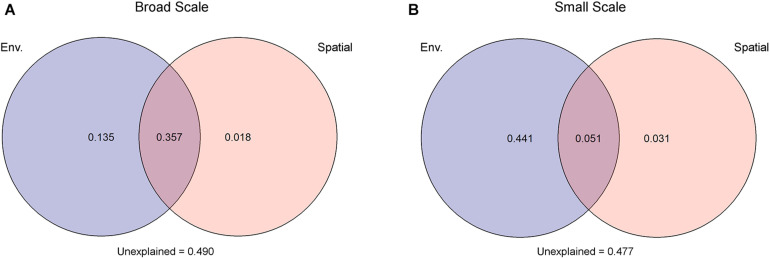
Variation partitioning analysis attested that environmental factors (Env.) had a greater impact on the observed differences in mycoplankton assemblages than spatial factors. This holds true under two spatial scenarios: **(A)** broad scale and **(B)** small scale. Spatial scenarios are based on a scalogram using the Moran’s I coefficient as ordinate, where significant distance-based Moran’s eigenvector map (dbMEM) eigenfunctions were defined to the two scaling submodels (see Supplementary Figure 6).

Due to collinearity among some of the environmental variables (Supplementary Table 2), three distant models were tested. The best model identified salinity (F = 9, *P* < 0.001) and PO_4_ (F = 3.5, *P* < 0.01) as most important environmental factors, which were then used as input for VPA. For both spatial models applied in VPA, environmental parameters explained more of the observed variability than spatial factors. The model with a broad-scale effect identified 49.2% of the variations to be under environmental control, but the larger part (73%) of the environmental-dependent variation was shared with spatial factors pointing toward a spatial control of the environment. Only 1.8% of the observed variation among fungal communities was under spatial control alone, while 49% of the observed variation stayed unexplained. In the small-scale model, 49.2% were explained by the environment from which 10% were under spatial control. Spatial factors alone explained only 3.1% of the observed variations; 47.7% stayed unexplained (Figure 3). The two other environmental models, with (I) chlorophyll *a* (*F* = 4, *P* = 0.001), DOC (*F* = 6, *P* = 0.001), and NO_3_ (*F* = 3, *P* = 0.016), or (II) temperature (*F* = 7, *P* = 0.002) and PO_4_ (*F* = 6, *P* < 0.001) as most driving factors, were tested with the spatial models but showed similar trends. Thus, environmental factors explained always a higher percent of the observed variation than spatial factors (Supplementary Figure 7).

### Distant Assemblage Processes Dominate Different Sampling Sites

**FIGURE 4:**
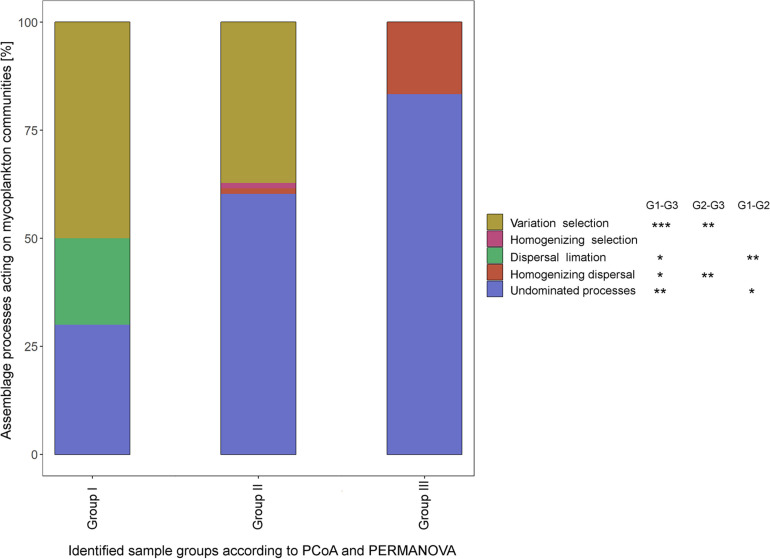
Different assemblage processes within the three sample groups as defined by quantitative process estimates (QPEs) method. Significant differences in the underlying assemblage processes between sample groups was tested by Tukey honestly significant difference (HSD) (*P < 0.05; **P < 0.01; ***P < 0.001).

QPE identified five different assembling processes acting on the fungal communities, namely, variation selection, homogenizing selection, dispersal limitation, homogenizing dispersal, and undominated processes. While variable selection dominated with 50% of the assembly processes in the upstream regions (group I) of the Elbe, undominated processes became dominant (60.3%) in the sample group II (dredged section of the Elbe) and group III (outer rive plume and beyond, 83.3%). All but homogenizing selection showed significance between samples group I and III (Tukey HSD, *P* < 0.05), while variable selection and homogenizing dispersal differed significantly among sample groups II and III (*P* < 0.01) and dispersal limitation and undominated processes among sample groups I and II (*P* < 0.05) (Figure 4).

### Salinity as Driving Factor for Distribution of Basal Fungi Within the Water Types of Marine, Brackish, and Freshwater

**FIGURE 5:**
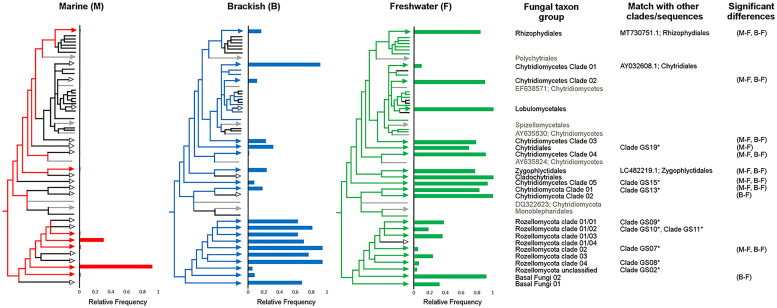
Relative frequencies of basal fungal taxon groups within the three water types of fresh, brackish, and marine waters. Partial sketch of the phylogenetic tree showing the relationship of the basal fungal lineages (for further details on the naming of the environmental clades, see Supplementary Figure 1, and for the original tree, see Supplementary Material 2). Colored branches indicate the presence of the specific taxon group in the given water type; black branch indicates absence. Significant differences in frequency between water types based on Tukey honestly significant difference (HSD) *P* values (*P* < 0.05). Neighbor taxa to the clades in the phylogenetic tree, not originating from the transect, are shown in gray. The column “Match with other clades/sequences” indicates the accession number of sequences with high BLASTn scores with sequences of in this study newly identified environmental clades and/or indicates that representative sequences from Tedersoo et al. (2017) identified clades were phylogenetically placed on the branch of the environmental clades identified in this study.

Significant differences were observed in the frequency of the basal fungal taxa over the three water types of marine, brackish, and freshwater. The majority of Chytridiomycota taxa (12 taxa, 92%) were most frequent in freshwater. Among the eight Rozellomycota clades, seven were most frequent in brackish water. None of the taxa were only present in the marine environment. Most of the observed significant differences were found between marine and freshwater water types, to a less extent between fresh- and brackish waters, and only one between marine and brackish waters (Tukey HSD, *P* < 0.05) (Figure 5).

## Discussion

In order to develop sustainable concepts for river systems, where human use harmonizes with the stability of the ecosystem, it is important to have knowledge on the diversity and dynamics of central ecosystem key players. Pelagic fungi occupy a central role in the carbon cycle and food webs of freshwaters (Wurzbacher et al., 2010; Grossart et al., 2019). In contrast to other lotic systems (Krauss et al., 2011; Monchy et al., 2011; Duarte et al., 2015; Lepere et al., 2019), the mycoplankton of streams with higher order number is a poorly studied group of organisms. In this study, for the first time, mycoplankton communities were surveyed with fungus-specific primers and high-throughput sequencing over a transect encompassing the shallow freshwater area up to the river plume of the Elbe River and beyond to the marine area. This study aims to provide an initial picture on mycoplankton community pattern and underlying assemblage processes in order to implement more targeted studies, in which seasonality and different degrees of water runoff should be included. However, despite the short sampling time, the results reflected the site-specific conditions of the investigated Elbe section: Thus, in the Elbe, the water body is exposed to strong environmental changes during its passage through the riverbed, which many of them are (indirectly) associated with anthropogenic activities (Schöl et al., 2014; Geerts et al., 2017; Sanders et al., 2018). Thus, the effect of the most structural variable, the salinity gradient, was partly superimposed by secondary effects of Elbe deepening works, especially downstream of the city of Hamburg. As a consequence, the patterns of mycoplankton communities deviated from a clear grouping into marine, brackish, and freshwater communities as shown for bacterioplankton in other river systems (Selje and Simon, 2003; Henriques et al., 2006). Mycoplankton communities formed three distant biomes, in which Dikarya and basal fungi showed opposite distribution patterns with a dominance of Chytridiomycota in the upstream and estuary sections of the Elbe toward a dominance of Ascomycota in the end members of the river plume and in marine waters. In addition to environmental parameters, however, spatial parameters can also be important drivers of microbial community structures. River systems, for example, support passive organismic dispersal, from which pelagic microorganisms with small body size benefit much more than larger, multicellular organisms (Soininen et al., 2013). In this study, the phylogenetic similarity of mycoplankton communities declined significantly with increasing distance between sampling sites. However, the VPA determined that environmental factors, to some degree under spatial control, had the greatest influence on the mycoplankton community structure. Thus, it may be concluded that the process of fungal assemblage across the sampled transect depended largely on niche-based rather than neutral processes. However, niche-based processes cannot always be explained by environmental filtering alone, as selection pressure increases or decreases along the environmental gradient (Stegen et al., 2012). In the latter case, other processes such as low dispersal rates can still lead to a high taxon turnover between sites and evoke separation of communities (Stegen et al., 2013; Vass et al., 2020). This shows that multiple processes can simultaneously govern ecological systems. Especially for a naturally nested system like rivers, it is even more important to distinguish between assemblage processes resulting from a combination of variable selection linked to low or high dispersal rates, which is why the ecological framework of Stegen et al. (2015) was applied in this study. This distinguishes between five different assemblage processes, namely, variable selection, homogenizing selection, dispersal limitation, homogenizing dispersal, and undominated process like ecological drift. Applied to the studied Elbe transect, significant differences were observed: Variable selection dominated in the upstream samples, whereas the proportion of undominated processes, where neither selection nor dispersal dominated, increased toward the river mouth and beyond.

Sample group I comprised all samples of the shallow freshwater area upstream of the city of Hamburg, which is a highly saturated eutrophic system, characterized by strong phytoplankton growth with a seasonal mean of 150 μg chlorophyll *a* L^–1^ and an oxygen oversaturation of the water (Schöl et al., 2014). The strong growth and high diversity of phytoplankton are a source for a rich plankton community forming complex food webs (Kerner et al., 2004). The mycoplankton communities within group I were dominated with up to 84.6% of the relative sequence abundance by Chytridiomycota. Taxa in this phylum are saprotrophs, or pathogens infecting phytoplankton, zooplankton, insects, and other fungi (Gleason et al., 2008). Both nutrition modes were found among the detected Chytridiomycota OTUs. Their ecological versatility makes them key players in food web dynamics affecting primary producers, predators, grazers, and degraders (Gleason et al., 2014; Kagami et al., 2014). The high taxon richness and phylogenetic diversity detected in this study suggest that they occupy diverse ecological niches and are involved at various trophic levels of the food webs established in this Elbe section. The often specific interactions among host–parasite or prey–grazer (Holfeld, 1998; Pinto et al., 2001; Ibelings et al., 2004; Vargas et al., 2006) may be among the reasons why variable selection was the dominant assemblage process in this sample group. Dispersal limitation was the second most important one. A major factor is certainly the weir at the level of Geesthacht, which prevents tidal upstream mixing of water between samples 3 and 4. Factors causing a dispersal limitation of microorganisms have been further identified on a microscopic level (Peay et al., 2010; Stegen et al., 2013). This is the case when local conditions inhibit subsequent colonization from one niche to another, such as reported for cells from particle-associated biofilms inhibited to colonize the surrounding water (Martiny et al., 2011). The extent to which such barriers play a role in the assembly of mycoplankton within the studied Elbe River transect needs to be investigated in further works.

Interestingly, the mycoplankton communities found downstream from the city of Hamburg differed significantly from sample group I, although some sample sites still had a salinity value within the freshwater range. From the port of Hamburg, the environmental conditions change drastically due to a sudden increase in depth to a maximum of 15 m caused by dredging (Geerts et al., 2017). The consequence is light limitation and strongly reduced oxygen content, while the net phytoplankton growth becomes negative and the system changes from an autotrophic to a heterotrophic one (Amann et al., 2012; Schöl et al., 2014). The significant separation of sample groups I and II, which were both dominated by Chytridiomycota, was partly due to abundance shifts of existing OTUs together with a strong reduction in phylogenetic diversity. However, one-third of the observed beta-diversity among sites could be attributed to taxon turnover by variable selection. The presence of numerous OTUs over larger sections of the estuary and the increase in abundance of various OTUs at different sample locations suggest a niche-specific growth and rules out that these OTUs are only inoculum-like resting spores. This observation also speaks against the possibility that the abundant OTUs are sediment-inhabiting taxa flushed up by turbulences. The residence time of the water body during summer time with low discharge is up to 11 days in the first 30 km downstream from Hamburg and even up to 35 days in the areas further downstream to the mouth of the Elbe, which is also a consequence of the Elbe dredging (Bergemann et al., 1996; Amann et al., 2012). This may give the mycoplankton communities time to grow in their specific niche and/or react to site-specific conditions. Some of the most abundant Chytridiomycota OTUs in the Elbe estuary were identified as saprotrophs and showed significant correlation with DOC. In the estuary of the Elbe River, there are various inputs of organic substrate, from external sources, including marine-, terrestrial-, and river-derived algae detritus and wastewater, and *in situ* estuarine sources (Middelburg and Herman, 2007). Chytridiomycota are among the important players of freshwater degradation (Sparrow, 1960; Wurzbacher et al., 2010) and have been reported to decompose smaller particles or benefit directly from DOC (Wurzbacher et al., 2014). By considering the frequency at sampling sites and the distribution pattern along the estuary of individual Chytridiomycota OTUs, three different scenarios can be deduced. First, the potential saprotrophic taxa possess a broad substrate specificity (Gleason et al., 2011) and/or are actors within functional guilds. Second, they may occupy specific niches that are maintained over longer stretches of the estuary through a long residence time. This may especially hold true for sample locations 8–13, which are situated in a river section with high particulate organic carbon (POC) concentrations (Amann et al., 2012). These first two scenarios may be reflected in continuous OTU abundance over four and more adjacent sampling sites as reported for several abundant OTUs. Third, taxa with pronounced abundance at single sampling sites may have proliferated quickly because the appropriate food source, such as labile organic material, was available. The percentage of labile POC in rivers increases, for example, through the input of sewage (Etcheber et al., 2007), as it occurs in the Elbe estuary due to the strong anthropogenic activities of the Hamburg city area. Beside variable selection, mycoplankton communities in group II were largely assembled by ecological drift, a process, where moderate dispersal rates are coupled with low selection pressure. Ecological drift causes taxon abundances to vary, lowering diversity within communities and increasing differences among otherwise similar communities. One process affecting drift in natural systems are indirect multispecies interactions (Gilbert and Levine, 2017), and their level of complexity has a profound impact on the assembly of eukaryotic microbial communities (Bock et al., 2020). According to Read et al. (2015), microbial networks are particularly complex in downstream sites because the river water is older and contains a planktonic community that is in a later stage of ecological succession. Banos et al. (2020) recently showed that a large proportion of taxa in marine pelagic mycoplankton taxa interact with each other in manifold ways such as in competition or in potential functional guilds. Applied on the Elbe transect, this situation may be particularly true for the areas around the mouth of the river with the longest water residence times.

Sample group III included samples in the area, where the river plume faded out and conditions became marine. In this area, the Elbe River outflow mixes with ocean waters due to western incoming currents, and the hydrographic conditions change to oceanic (Callies and Scharfe, 2015). Lucas et al. (2016) showed that this leads to a significant difference between bacterioplankton communities in the inner German Bight and those in the open North Sea. The authors assumed Helgoland to be the eastern boundary of the main current direction in the German Bight. Relative to the mycoplankton communities, the lowest phylogenetic diversity and taxon abundance was found in group III. Additionally, communities were now dominated by Ascomycota. A comparison with the mycoplankton communities monitored over a full year at the nearby long-term research station Helgoland Roads (Banos et al., 2020) showed large agreement with the communities of samples 20-24. In the mentioned study, mainly Ascomycota dominated the mycoplankton communities. The observed significant taxonomic shift between groups II and III may, thus, have resulted from the interplay of different converging processes: (I) Growth and reproduction of pelagic Chytridiomyota seems to be favored in environments with lower salinities compared to the open oceans (Hassett and Gradinger, 2016; Hassett et al., 2019). Bouvier and del Giorgio (2002) reported a similar abrupt change between salt-tolerating and less tolerant bacterioplankton communities relating it to organismic salt tolerance resulting into cell inactivation or cell death. (II) The negative response of some phytoplankton species to marine salinity values (Lionard et al., 2005; Nakov et al., 2019) has, in consequence, a negative effect on the presence and abundance of the associated parasitic Chytridiomycota taxa. (III) One of the significant assemblage processes for mycoplankton communities in group III was homogenizing dispersal. This may point toward an increased input from upstream regions of the Elbe River, which may include (terrestrial) Ascomycota taxa that are able to proliferate in the marine environment. However, it has to be noted that marine fungal communities can be dominated by zoosporic fungi (Comeau et al., 2016). Studies from the North Sea reported temporary dominance (Priest et al., 2021) and showed, e.g., dependence on host abundance and nutrient availability (Scholz et al., 2016).

Despite the different processes acting on the mycoplankton communities, our model showed that the salt gradient was the most important environmental parameter for the structuring of the mycoplankton community. In river estuaries, salt gradients are of general relevance, as they often define structural and functional characteristics of aquatic biota (Telesh and Khlebovich, 2010). In numerous studies, the value around 8 PSU has been described as a threshold value that significantly divides the organism groups under investigation (Sagert et al., 2008; Bleich et al., 2011; Schubert et al., 2011). This was also the case for mycoplankton communities that were studied along a salinity gradient of the Baltic Sea (Rojas-Jimenez et al., 2019). In our study, there was no group splitting at this threshold value. Only when specifically tested for the value of 8 PSU, a significant separation of mycoplankton communities could be observed. The Elbe is under strong tidal influence, and a salt wedge is formed, which is relatively inside the Elbe with a maximum at the level of Stade (samples 11 and 12). Along the salt wedge, there is an increased exchange of fresh and ocean waters. As side effect, marine particulate organic matter can be found in all group II samples (Schöl et al., 2014). Thus, a whole bouquet rather than a single factor impacts on the mycoplankton community structure, preventing a split of communities at 8 PSU in a multivariate approach. In our study, the most pronounced and significant change in the mycoplankton community structure was registered at a threshold level of 30 PSU, while all brackish water samples with their existing variability formed a significant group. Brackish-water-specific niche formation was already detected, for example, for fluvial bacteria (Selje and Simon, 2003) and fungi in salt marshes (Mohamed and Martiny, 2011). The protistan species-maximum concept developed by Telesh et al. (2013) encapsulates this observation and shows that unicellular eukaryotes are particularly strongly represented in the horohalinicum zone. One reason for this is that biota from two adjacent sites are mixed. Furthermore, for many organisms, the limiting factor is not the organism’s own salinity tolerance but the salinity variability (Attrill, 2002; Telesh et al., 2011). Most eukaryotic unicellular planktonic organisms have a high physiological adaptation to fluctuating salinity, and therefore, the salt content provides subsidy rather than a stressful environment (Stock et al., 2002; Telesh et al., 2011). Thus, they can develop in a system with reduced competitive pressure. Chytridiomycota belong to this group with a small body size of 3–5 μm as zoospores (Gleason and Lilje, 2009). The most abundant OTU, Chytridiomycota SMBZZZZ9, was found in almost all samples and thus spanned a salt gradient from 0 to 31 PSU. However, this is probably rather an exception. Gleason et al. (2006) tested numerous Chytridiomycota for their salt tolerance. Most of them showed growth success in salt-containing medium mimicking brackish but not ocean water salinity levels. The grouping of the samples along their salinity into the three water types of fresh, brackish, and marine waters revealed no true marine zoosporic fungal clade. Furthermore, each of the identified zoosporic fungal clades was found in at least two of the three water types, mostly in fresh and in brackish waters. The evolutionary history of Chytridiomycota suggests that they originated in a brackish-/freshwater-like environment (Berbee et al., 2017) and thus evolved successively in a series of transient brackish waters, where they were able to develop and diversify in parallel with the developing host organisms or existing food (Chang et al., 2015). The dependence of parasitic Chytridiomycota on the adaptation of the host organisms over the salt gradient as well as the reduced adaptability of most Chytridiomycota to high salt concentrations are two important points probably controlling for the presence and abundance of Chytridiomycota in fresh- and brackish water. It remains an open question whether marine Chytridiomycota proliferate due to lower competition in high-salinity water or whether specialization of some taxa has occurred over an evolutionary time scale but underwent so far detection.

## Conclusion

Understanding the relative importance of dispersal and environmental selection in shaping mycoplankton community in water may help predictions of fungal-driven ecological processes, like mycoflex, or mycoloop, and conservation of biodiversity in river ecosystems. Our results show that the mycoplankton communities in the Elbe River from the shallow freshwater zone over the estuary and its river plume are subjected to very different assemblage processes and differ significantly from those stations subjected to strong marine influence. Additionally, assemblage processes can change over relatively short distances. Community assembly processes are not static, and the relative importance of one can vary under different conditions and between members of a community. Further work is needed to understand how strongly the assembly processes observed here are related, for example, to the strength of the water runoff and which consequences a shift in the community composition has on the fungal-driven ecological processes.

## Data Availability Statement

The generated sequence datasets can be obtained from the European Nucleotide Archive (ENA) with the accession number PRJEB39018. The corresponding environmental data is published in PANGAEA (Reich et al., 2021). The fully annotated OTU table can be accessed over the Supplementary Table 4, representative sequences for each OTU over a Supplementary .fasta-file (Data Sheet 2), and the phylogenetic tree including the inserted generated sequences over a Supplementary .tree-file (Data Sheet 3).

## Author Contributions

MR, GG, and AW planned and designed the study. YY and SB ran the sequencing pipeline. YY, GG, and MR analyzed the data. YY, SB, and MR wrote the manuscript. All authors have reviewed and approved the manuscript.

## Conflict of Interest

The authors declare that the research was conducted in the absence of any commercial or financial relationships that could be construed as a potential conflict of interest.

## Footnotes

1 https://github.com/stegen/Stegen_etal_ISME_2013
