# Supplementary material for: Mycoplankton Biome Structure and Assemblage Processes Differ Along a Transect From the Elbe River Down to the River Plume and the Adjacent Marine Waters
Source: Front Microbiol. 2021 Apr 23;12:640469. doi: 10.3389/fmicb.2021.640469 (PMC8102988; doi:10.3389/fmicb.2021.640469)
Supplement: Supplementary file 1 [file Data_Sheet_1.PDF]

Supplementary Material

Myoplankton biome structure and assemblage processes differ significantly along a transect from the shallow freshwater area of the Elbe River down to the river plume and the adjacent marine waters

Yanyan Yang, Stefanos Banos, Gunnar Gerdts, Antje Wichels, Marlis Reich

1.1 Supplementary Figures

Supplementary Figure S1. Sketch of the fungal phylogenetic tree to explain why environmental clades were classified at different taxonomic levels. Novel diversity clades are colored in blue. Their taxonomic position depends on the branch on which the clade is located and if this branch has a reliable taxonomic assignment.

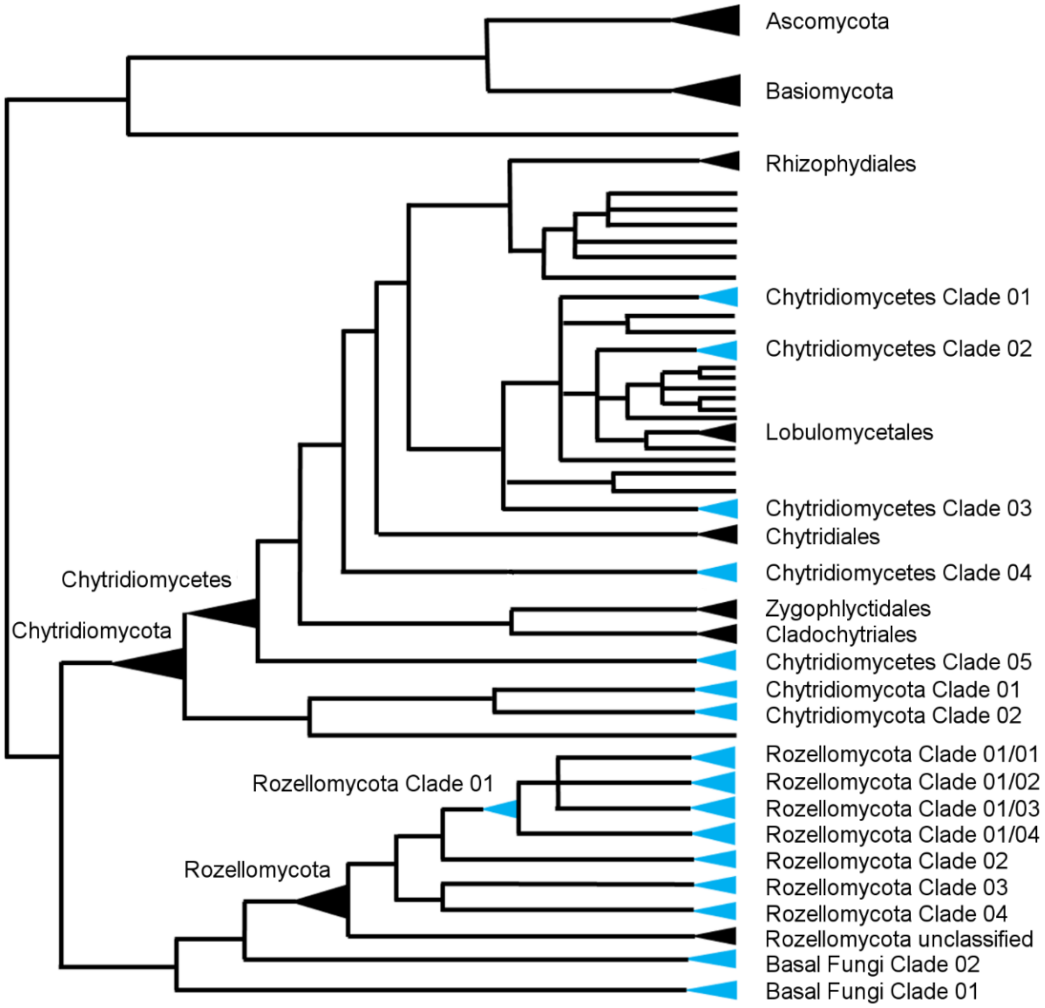

Supplementary Figure S2. Rarefaction curves calculated on all generated sequences (communities over the total transect) and sample group wise (see PCoA, Fig. 2). Shaded area indicates the 95%-confidence interval.

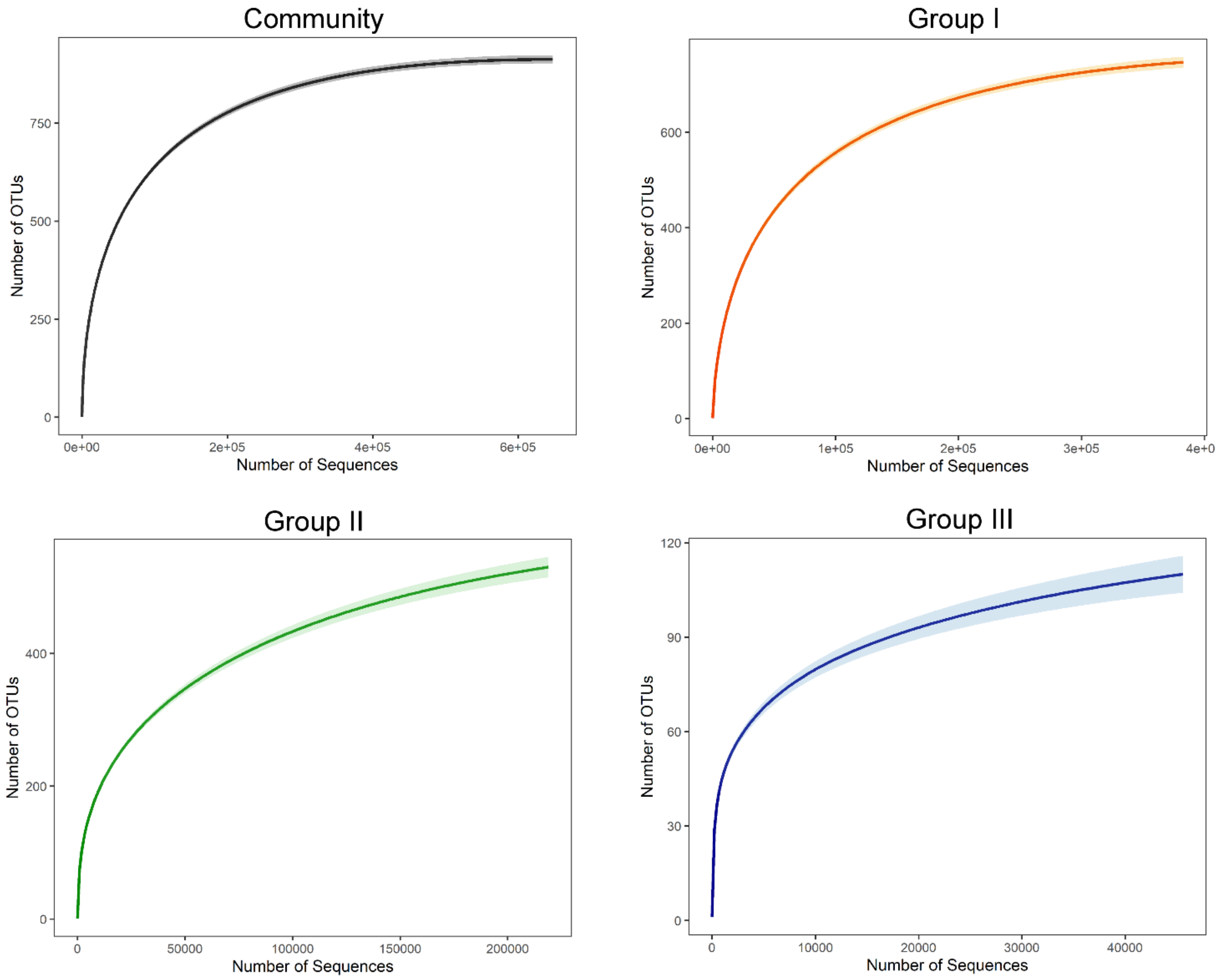

Supplementary Figure S3. Within the estuary of the Eble River, two different distribution patterns of the abundant OTUs were observed (samples 7-24). OTUs being present (A) over numerous adjacent sampling sites (>4; like OTU SMBZZZ14, Rhizophydiales, saprotroph), or (B) high frequency at single sampling sites if occurring in adjacent sites in a maximum of 3 adjacent sampling stations (like OTU SBMZZZ18, Chytridiomycota clade 01, saprotroph).

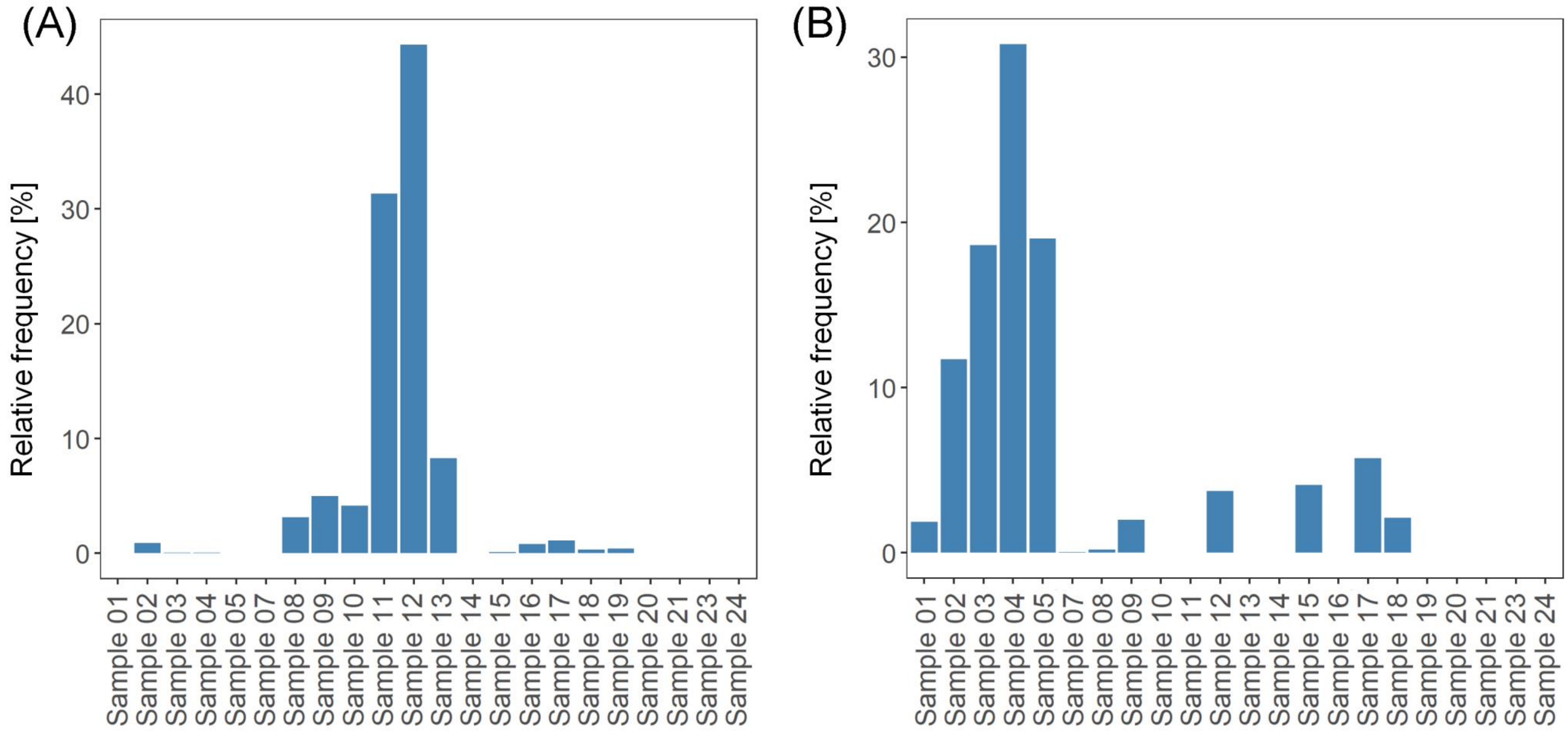

Supplementary Figure. S4: PCoA segregating the mycoplankton communities of the end members of the studied transect (marine environment, samples 20-24) with mycoplankton communities of Helgoland Roads described by Banos *et al.* (2020). PCoA is based on Generalized UniFrac values with the distance parameter of "0" accounting only for the phylogenetic structure and not abundances. Samples 1-24 are samples from the transect. Red, group I; green, group II; blue, group III. Group III are marine samples where the river plume faded off. Samples 45-87 in pink are from Helgoland Roads (Banos *et al*., 2020, DOI: 10.3389/fmicb.2020.01305).

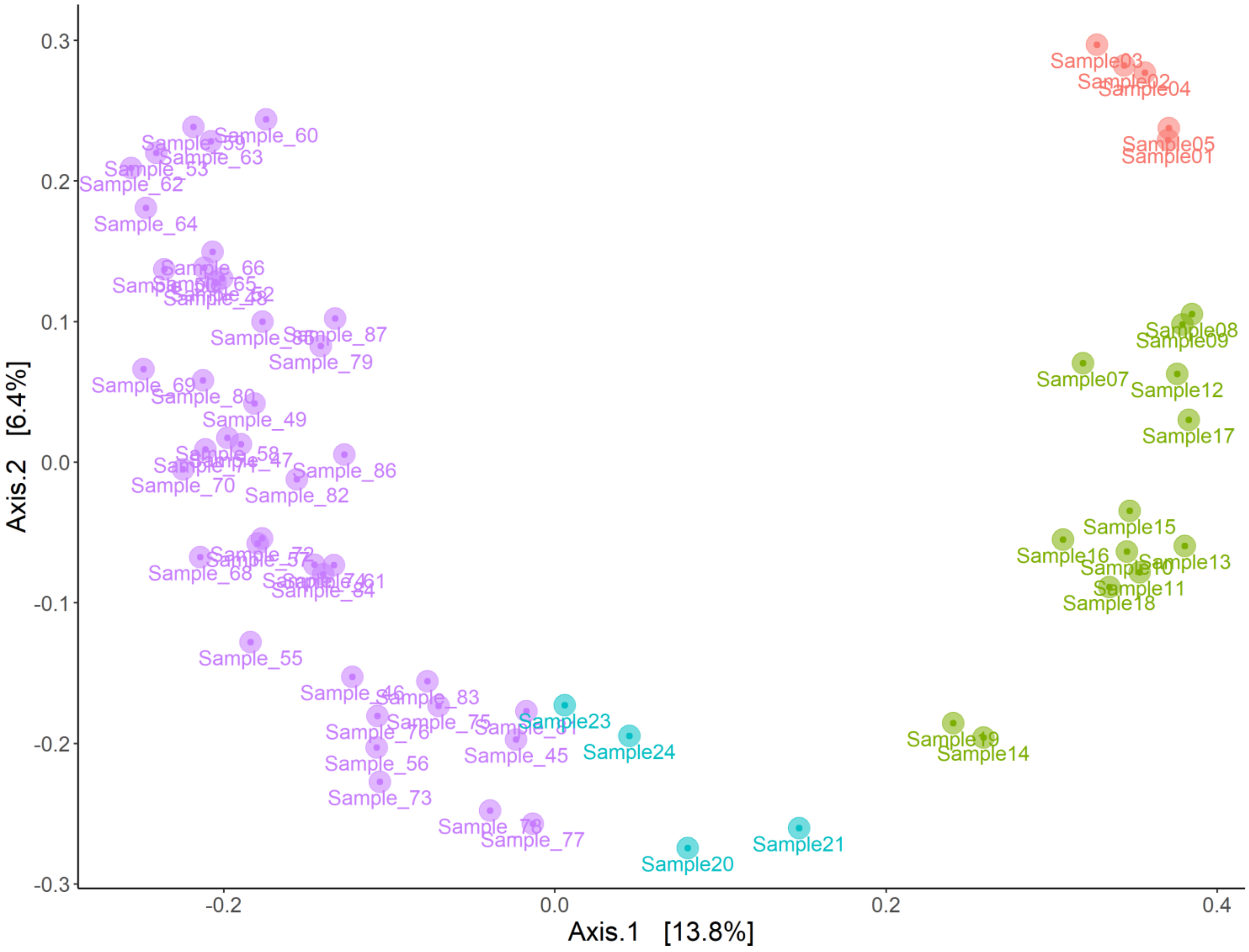

Supplementary Figure S5: Distance-decay analysis based on linear regression indicating a significant relationship of increasing phylogenetic dissimilarity among communities with increasing distance (km) of sampling sites. Phylogenetic dissimilarity calculated as Generalized UniFrac-value. Distance between sampling sites was calculated as cumulative water channel distance.

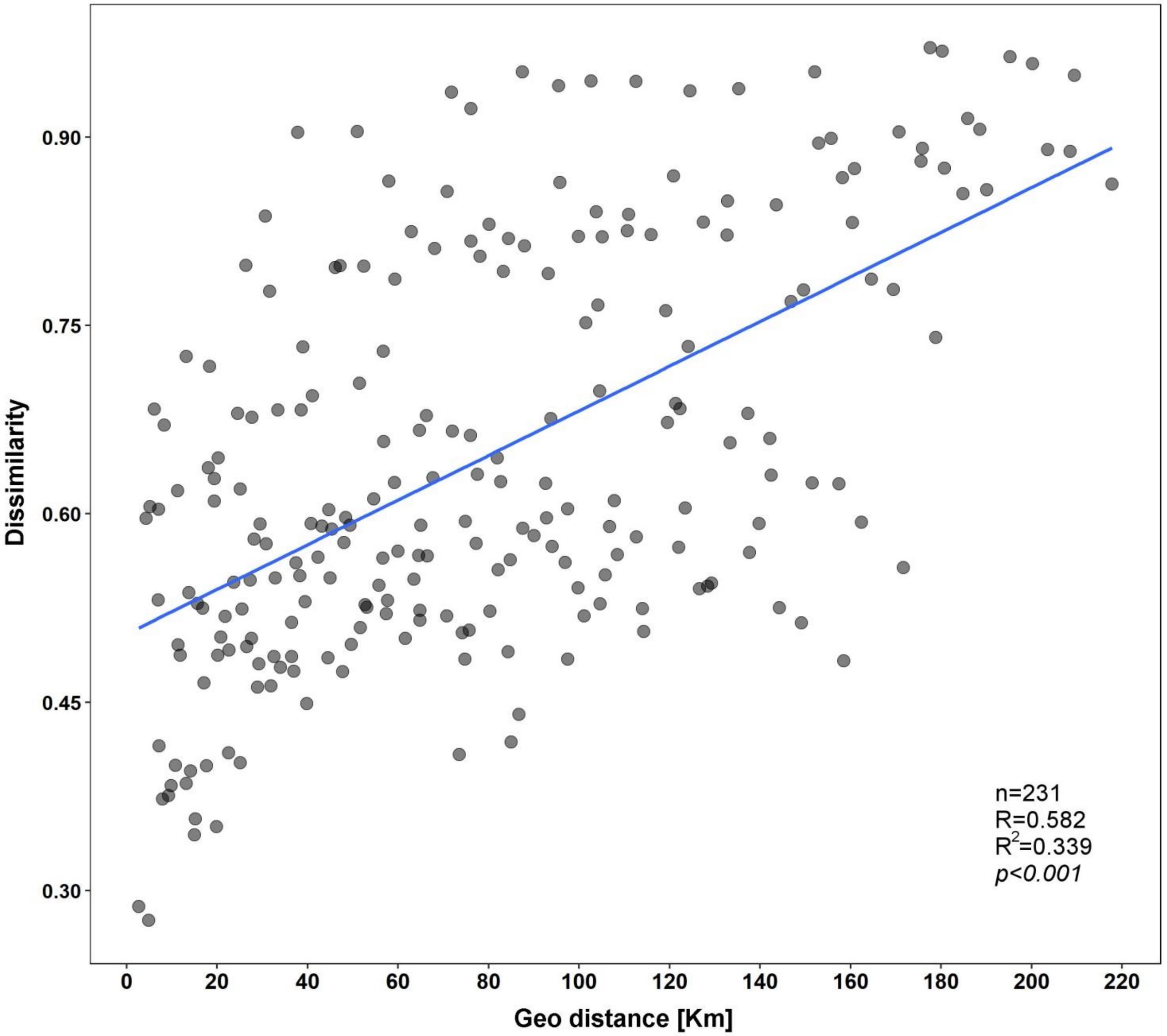

Supplementary Figure S6. Definitions of the two spatial submodules used for VPA analysis were based on the scalogram using the Moran's I coefficient as ordinate. *, significant eigenfunctions (forward selection, $P < .05$).

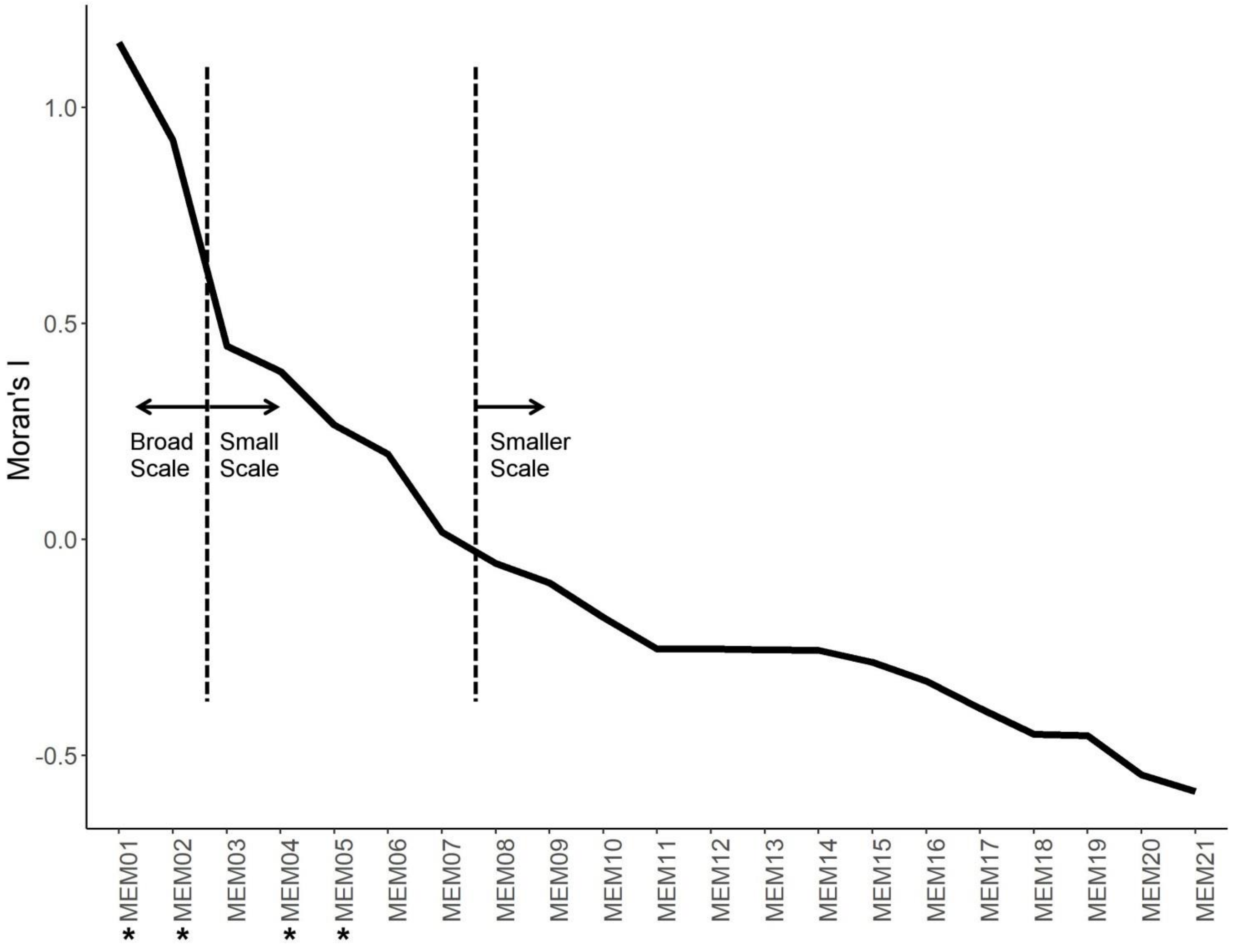

frontiers

Supplementary Figure S6: VPA calculated with three different environmental models. Due to high collinearity among some of the environmental variables, three different models were calculated with db-RDA based forward selection. The best model was the one with salinity and PO_4. However, all models showed a similar trend indicating that observed variability among mycoplankton communities in the lower reaches of the Elbe River are mainly driven by environmental factors, which are partly under spatial control.

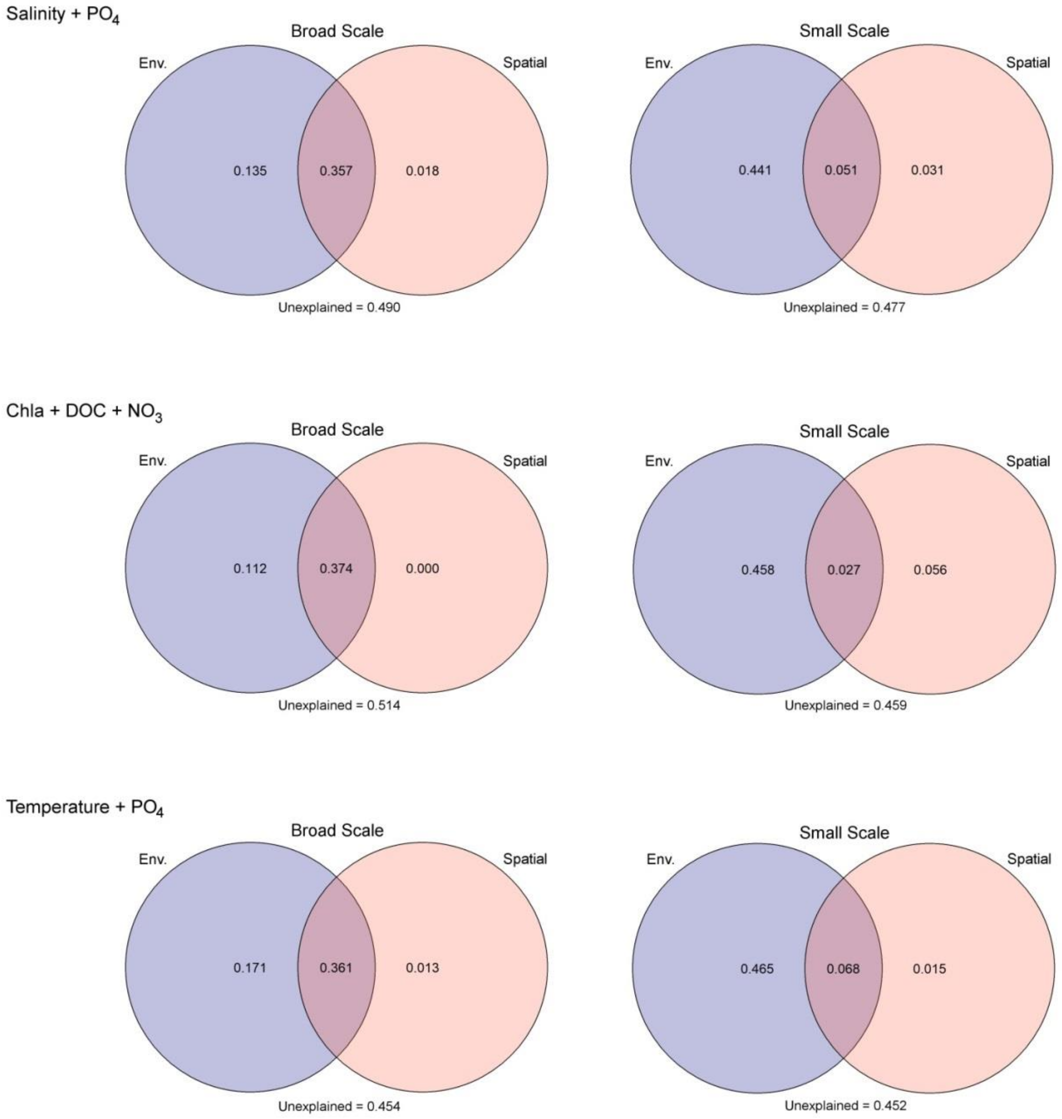

1.2 Supplementary Tables

Supplementary Table S1: Geographic location and grouping of samples. Sample grouping as given by PCoA and confirmed by PERMANOVA analysis ($P < 0.05$). Additionally, samples were manually grouped into samples of fresh, brackish, and marine water types based on their salinity value. Some samples were spared out from this analysis as they strongly vary in salinity over the course of a year and thus cannot be assigned into one of the groups based on water types (see materials and methods).

Sample	Ost	Nord	Temp. (°C)	DOC (µmol/l)	Chl *a* (µg/l)	SiO4 (µmol/l)	PO4 (µmol/l)	NO2 (µmol/l)	NO3 (µmol/l)	NH4 (µmol/l)	pH	Salinity (PSU)	Depth (m)
Sample1	10.552	53.370	24.2	432.22	91.58	60.42	0.34	1.03	43.22	1.84	8.94	<0.5[$]	2.50[*]
Sample2	10.427	53.401	24.7	417.63	68.06	48.05	0.09	0.87	33.74	1.18	9.07	<0.5[$]	2.50[*]
Sample3	10.367	53.428	24.1	422.77	72.04	49.90	0.19	1.23	51.72	1.60	8.92	0.41[#4]	2.28[*]
Sample4	10.173	53.395	22.2	365.34	55.69	88.40	0.24	1.16	56.80	2.74	8.34	0.43[#3]	3.50[*]
Sample5	10.143	53.415	22.3	413.65	56.74	62.71	0.36	0.96	46.37	2.85	8.54	<0.5[$]	3.50[*]
Sample6	9.984	53.474	20.9	409.12	50.37	46.63	0.31	0.93	41.05	3.61	8.31	NA	NA
Sample7	9.879	53.536	21.2	472.51	15.42	30.18	1.49	2.46	74.28	4.30	7.81	0.47[#2]	7.00[*]
Sample8	9.633	53.573	20.3	399.20	5.18	33.77	1.93	0.10	64.17	0.76	7.76	0.49[#1]	7.00[*]
Sample9	9.517	53.643	20.0	387.59	5.39	39.41	1.29	0.09	99.79	0.40	7.78	0.70	19.50[*]
Sample10	9.431	53.733	20.0	401.15	5.88	49.48	1.48	0.11	103.60	0.48	7.87	1.00	13.00[*]
Sample11	9.363	53.813	19.0	567.21	6.13	54.23	1.72	0.27	107.73	0.41	7.99	2.00	20.00[*]
Sample12	9.287	53.860	19.0	418.66	3.79	54.43	1.83	0.72	104.69	0.72	7.96	4.20	15.00[*]
Sample13	9.168	53.877	18.0	422.97	4.17	52.35	2.25	1.19	95.58	0.48	7.96	7.40	15.00[*]
Sample14	8.998	53.850	18.0	399.67	6.45	36.94	1.69	1.07	75.40	1.84	8.03	12.90	7.00[*]
Sample15	8.935	53.842	18.0	354.37	10.39	30.67	1.79	0.97	67.28	2.63	8.05	15.10	17.00[*]
Sample16	8.780	53.923	18.0	331.77	7.29	13.54	1.03	0.59	37.30	1.35	8.16	22.30	25.00[*]
Sample17	8.680	53.900	18.3	513.15	8.20	25.66	2.29	0.95	36.80	1.86	8.09	17.79	15.00
Sample18	8.500	53.950	18.1	315.26	6.62	9.64	1.31	0.59	18.79	1.44	8.17	27.03	13.00
Sample19	8.405	53.982	18.0	469.94	6.58	5.24	0.78	0.37	10.44	0.59	8.21	27.56	18.00
Sample20	8.312	53.990	18.1	280.26	4.62	4.00	0.46	0.20	4.07	0.25	8.18	29.88	18.00
Sample21	8.238	54.008	18.0	218.09	4.25	3.87	0.39	0.18	3.64	0.16	8.19	30.25	20.00
Sample22	8.083	54.050	17.8	177.19	3.48	4.39	1.38	0.18	3.00	0.83	8.20	30.22	20.00
Sample23	7.987	54.102	17.3	219.15	3.30	7.77	0.42	0.25	1.95	1.19	8.16	30.10	27.00
Sample24	7.892	54.152	17.6	179.61	2.73	5.04	0.17	0.17	1.26	0.12	8.22	31.35	53.00

#: values of the data portal "Fachinformationssystem (FIS)" of the FGG (FlussGebietsGemeinschaft, Magedeburg, Germany) Elbe; [1-4]distances from sample location of the FGG to sample location of this study:[1]0.6km, [2]0.5km, [3]0.2km, [4]1.5km
$: based on literature values: (Amann *et al*., 2014, DOI: 10.1007/s10533-013-9940-3; Carstens *et al*., 2004, DOI: 10.1002/aqc.652; Magath *et al*., 2013, DOI: 10.1111/jfb.12115)
*: using the digital relief model of the river from the Zentrales Datenmanagement (ZDM) of the Wasserstraßen- und Schifffahrtsverwaltung des Bundes

Supplementary Table S2: Collinearity of environmental parameters tested by Spearman rank order correlations. Only significant correlations (FDR adjusted $P < .05$) with high relevance ($R^2 \geq 0.5$) are shown. R, R^2-values are indicated in the table.

	T	DOC	Chl *a*	SiO_4	PO_4	NO_2	NO_3	NH_4	pH	Salinity
DOC										
Chl *a*										
SiO_4										
PO_4										
NO^-_2										
NO^-_3										
NH^+_4			0.73,0.53			0.75,0.56				
pH					-0.82,0.68					
Salinity	-0.92,0.84			-0.83,0.69						
Depth	-0.81,0.65		-0.72,0.52							0.83,0.68

Supplementary Table S3: Taxonomic classification and trophic mode of the most abundant OTUs. Representative sequences of the OTUs were inserted into the phylogenetic reference tree using phylogenetic placement. Taxonomy of the branch where sequences were placed, were transferred on the OTU. Additionally, BLASTn was carried out and best BLAST hits are reported. Based on the classification over BLASTn, literature was screened for information on taxa specific trophic modes.

-> See additional .xlsx-file

Supplementary Table S4: Fully annotated OTU table. The table provides frequencies of OTUs and detailed taxonomic information from the phylogenetic tree. Representative sequence for each OTU can be found in the attached .fasta-file Supplementary File S1.

->See attached .xlsx-file

Supplementary Table S5: Correlation analyses of the abundant OTUs with environmental parameters using Pearson rank order correlations. Only significant correlations (FDR adjusted $P < .05$) are shown. R, R^2 values are shown in the table.

OTUs	Phylum	Temperature	DOC	Chl *a*	SiO_4	PO_4	NO_2
SMBZZZ15	Ascomycota		-0.43, 0.18		-0.45, 0.20	-0.50, 0.25	
SMBZZZ13	Ascomycota				-0.68, 0.46		-0.51, 0.26
SMBZZZ16	Ascomycota				-0.54, 0.29		-0.49, 0.24
SMBZZZ10	Chytridiomycota	0.71, 0.50		0.67, 0.45	0.67, 0.45		0.46, 0.21
SMBZZZ11	Chytridiomycota	0.63, 0.40		0.50, 0.25	0.76, 0.58		0.42, 0.18
SMBZZZ14	Chytridiomycota		0.47, 0.22			0.55, 0.30	
SMBZZZ23	Chytridiomycota	0.72, 0.52		0.70, 0.49	0.54, 0.29		0.44, 0.19
SMHZZZZZ	Chytridiomycota	0.77, 0.59	0.44, 0.19	0.43, 0.18	0.73, 0.53		
SMBZZZ17	Chytridiomycota		0.45, 0.20			0.74, 0.55	
SMBZZZ19	Chytridiomycota	0.58, 0.34		0.63, 0.40	0.46, 0.21		0.44, 0.19
SMBZZZ21	Chytridiomycota	0.69, 0.48		0.76, 0.58	0.43, 0.18		0.44, 0.19
SMBZZZ12	Chytridiomycota	0.89, 0.79	0.54, 0.29	0.79, 0.62	0.70, 0.49		
SMBZZZZ9	Chytridiomycota	0.57, 0.33	0.49, 0.24	0.43, 0.18	0.62, 0.38		
SMHZZZZ2	Chytridiomycota	0.61, 0.37		0.43, 0.18	0.65, 0.42		
SMHZZZZ3	Chytridiomycota	0.83, 0.69		0.46, 0.21	0.69, 0.48		
SMBZZZ18	Chytridiomycota	0.66, 0.44		0.62, 0.38	0.57, 0.33		0.48, 0.23
SMHZZZZ4	Basal Fungi clade 02	0.79, 0.62		0.58, 0.34	0.53, 0.28	0.67, 0.45	

Table is continued on the next page…

OTUs	Phylum	NO_3	NH_4	pH	Salinity	Depth
SMBZZZ15	A_Chaetothyriales	-0.79, 0.62		0.53, 0.28		
SMBZZZ13	A_Hypocreales	-0.58, 0.34	-0.47, 0.22		0.48, 0.23	
SMBZZZ16	A_Hypocreales	-0.57, 0.33			0.51, 0.26	
SMBZZZ10	Chytridiomycota		0.45, 0.20		-0.72, 0.52	-0.63, 0.40
SMBZZZ11	Chytridiomycota				-0.69, 0.48	-0.59, 0.35
SMBZZZ14	Chytridiomycota	0.63, 0.40		-0.47, 0.22		
SMBZZZ23	Chytridiomycota		0.54, 0.29		-0.75, 0.56	-0.64, 0.41
SMHZZZZZ	Chytridiomycota	0.47, 0.22			-0.80, 0.64	-0.53, 0.28
SMBZZZ17	Chytridiomycota	0.71, 0.50		-0.59, 0.35		
SMBZZZ19	Chytridiomycota		0.61, 0.37		-0.67, 0.45	-0.45, 0.20
SMBZZZ21	Chytridiomycota		0.62, 0.38		-0.70, 0.49	-0.61, 0.37
SMBZZZ12	Chytridiomycota		0.46, 0.21		-0.87, 0.76	-0.69, 0.48
SMBZZZZ9	Chytridiomycota	0.49, 0.24			-0.70, 0.49	-0.55, 0.30
SMHZZZZ2	Chytridiomycota	0.48, 0.23			-0.73, 0.53	-0.54, 0.29
SMHZZZZ3	Chytridiomycota	0.53, 0.28			-0.85, 0.72	-0.66, 0.44
SMBZZZ18	Chytridiomycota		0.59, 0.35		-0.66, 0.44	-0.67,0.45
SMHZZZZ4	Basal Fungi clade 02				-0.75, 0.56	-0.56, 0.31

Supplementary Table S5: Distance-based Moran's eigenvector (dbMEM) analysis identified seven eigenvectors with positive Moran I-values. Out of those, four were identified to have a significant effect on mycoplankton community structure as detected by dbRDA-based forward selection

	**MEM1**	**MEM2**	**MEM3**	**MEM4**	**MEM5**	**MEM6**	**MEM7**
Eigenvalues	0.204	0.164	0.079	0.069	0.047	0.035	0.003
Moran's I	1.15	0.924	0.448	0.389	0.265	0.197	0.017
**Forward selection on positive MEM factors**							
F-values	7.03	4.01		2.25	1.92		
P values	0.001	0.003		0.02	0.049		

Supplementary File S1: Representative sequences of all OTUs detected in this study. For detailed information on OTUs, see Supplementary Table S4.

-> see attached .fasta-file

Supplementary File S2: Phylogenetic tree as .tree-file. Phylogenetic reference tree (Yarza *et al.*, 2017), which was enriched with new fungal full length 18S rRNA gene sequences from the SILVA database. Next, generated sequences of this study were phylogenetically placed into the tree.

-> see attached .tree-file.
